# Monitoring Injuries Associated with Mandated Children’s Products in Australia: What Can the Data Tell Us?

**DOI:** 10.3390/ijerph15102077

**Published:** 2018-09-21

**Authors:** Kirsten Vallmuur, Caroline Lukaszyk, Jesani Catchpoole

**Affiliations:** 1 Institute of Health and Biomedical Innovation and School of Public Health and Social Work, Queensland University of Technology, Kelvin Grove 4059, Australia; jesanil@qisu.org.au; 2 Jamieson Trauma Institute, Metro North Hospital and Health Service, Royal Brisbane and Women’s Hospital, Herston 4006, Australia; 3 The George Institute for Global Health, University of New South Wales, Sydney 2042, Australia; clukaszyk@georgeinstitute.org.au

**Keywords:** child injury, injury surveillance, injury data, product-related injury, consumer product safety

## Abstract

Mandatory standard regulation is used within Australia to ensure the safety of consumer products, preventing product-related injury. Standard regulation is particularly important for products designed for use by children, who are highly vulnerable to sustaining product-related injuries due to their small size and inability to identify product hazards. This project aims to investigate how effectively information regarding product-related injuries is able to be captured within Australian health and coronial data. Further, it aims to investigate the extent to which child injury occurs for products for which mandatory safety standards exist through the review of available data. This study highlights significant limitations in injury surveillance data for identification and monitoring of child product-related injuries. This in turn limits the evidence base to assess the efficacy of existing regulations. Available data show baby walkers, cots, prams, nightwear, and bunk beds to be associated with a considerable number of child hospital presentations, admissions, and deaths. A significant scope for improvement in current product injury recording practices in the health sector exists.

## 1. Introduction

The consumer product safety environment is complex, involving a mix of regulating authorities, industry participants, front line clinicians, injury research professionals, community groups and consumers, all with different priorities and needs. Within Australia, the consumer product safety system covers all goods and product-related services that are not already covered by specific legislation or other regulatory bodies [1]. Product safety policy decisions have a major impact on all stakeholders, including industry and community. It is therefore important that policy decisions are well informed, logically argued, and clearly articulated to all stakeholders.

Product standards are widely used around the world to influence the safety of consumer products. Product standards are defined as published documents that specify the minimum requirements of a product’s specifications and outline procedures to ensure that the product can function safely for its purpose and consistently performs the way it was intended [1]. Safety standards outline safety requirements, with particular stipulations around performance, composition, contents, methods of manufacture or processing, design, construction, finish or packaging rules [2].

In Australia, mandatory standard regulation has been utilized by the national regulator to promote safety during the design and manufacturing stages of consumer products. This means that it is compulsory for the products under the regulation to be designed, produced, and packaged according to the related product standard (or part of the standard) for these products to be supplied legally in Australia [2]. This regulation also promotes hazard identification, risk assessment, and risk management at the design and manufacturing stage [1].

In Australia, a total of 41 products/product categories are regulated through mandatory standards, with almost two-thirds of these related to hazards for children. The full list of products with mandatory standards [3] includes 18 children’s products (including aquatic toys, baby bath aids, baby dummies, baby walkers, basketball rings/backboards, bunk beds, child restraints, folding cots, household cots, moveable soccer goals, nightwear for children, prams and strollers, projectile toys, self-balancing scooters, swimming and flotation aids, toys containing lead, toys containing magnets, and toys for children up to 36 months) and eight general products (including balloon blowing kits, bean bags, bicycle helmets, bicycles, exercise bicycles, novelty cigarette lighters, portable swimming pools, and treadmills) that are regulated due to the associated hazards posed to children. There are also other standards in place that are voluntarily utilized in Australia to define quality and safety criteria for products. However, it is still legal to supply products that do not meet voluntary standards.

In Australia, the information base to assess the frequency and severity of injuries related to mandated consumer products is limited, with evidence largely drawn from health and coronial data on product related injuries. The data lack consistency, particularly in relation to product causality in the injury (product failure, product use, product misuse). This limitation of the current evidence base was recognized in 2006 in a report into the Australian product safety system published by the Productivity Commission [1]. The report states (p. 359):

“*The available information on product-related injuries and deaths in Australia remains piecemeal, uncoordinated, and beset by methodological problems. As such, determining with any degree of precision the share of total injuries and deaths currently caused directly by unsafe consumer products, and trends in this share across time, is difficult.*”

This problem is not unique to Australia. The Organization for Economic Cooperation and Development’s (OECD) Working Party on Consumer Product Safety reported that critical knowledge gaps faced by regulators internationally include: the detection of unsafe products, enumerating the magnitude of the risk from actual incident data, identifying a suitable response, and communication across borders or stakeholders [4]. In the USA, there have been significant improvements to the transparency and availability of regulatory and injury data, with recalls, consumer complaints, and emergency department injury databases publically available for online searching and downloading of data files, providing a valuable resource for consumers, manufacturers and regulators alike [5]. However, in Australia, the Australian Consumer Law (ACL) included a strict confidentiality obligation on regulators receiving mandatory accident reports from suppliers; thus, only publically available and searchable data is on actual recalls. The ACL regime is also comparatively narrow in limiting the reporting requirement to actual incidents or rapid-onset illnesses or deaths, not other adverse health outcomes or serious risks (including ‘near misses’) [6].

In relation to product related injury events, current health and coronial systems are generally able to provide information on the product class (e.g., pram, bike, bed, chair, nail gun), demographics of the injured person (e.g., age, sex, location), injury mechanism (e.g., fall, struck by a moving object, crushing, suffocation, piercing, chemical, thermal), time of day of injury, part of the body injured and some level of detail of the injury event. The amount of detail provided and the amount of data captured depends on the data source. Coronial data provides a large amount of detail for a small number of fatal cases Australia-wide, hospitalization data provides limited but standardized information about causes of injuries Australia-wide, and specialized injury surveillance systems capture the product type and mechanism with more consistency, but only in a few jurisdictions.

The aim of this study was to (a) describe the extent to which health and coronial data capture information about products with mandatory standards pertaining to children and (b) where data are available for at least two of the three data sources (emergency presentations, hospitalizations, deaths), to describe the frequency and patterns of injuries and fatalities pertaining to mandatory standard regulated products in children.

## 2. Materials and Methods

### 2.1. Population

The population were all children aged 18 years and younger who have experienced a fatal or non-fatal product-related injury, captured through health or coronial data collection systems.

### 2.2. Data Sources

Data were obtained from three sources: the Queensland Injury Surveillance Unit (QISU), the National Hospital Morbidity Database (NHMD), and the National Coronial Information System (NCIS). QISU data is collected for all persons presenting with an injury or, in the case of children, from the accompanying adult, for a sample of emergency department (ED) presentations in Queensland. The data is estimated to cover approximately 25% of all ED presentations in in the state [7]. NHMD data provides information on all public and private hospitalizations in Australia on an episode basis and to account for multiple episodes of care for a patient (such as transfers to other facilities), cases with an admission mode of transfer were removed from analysis [8]. NCIS data contains details of all unexpected deaths and deaths resulting from accident or injury reported to the coroner for investigation in Australia [9].

### 2.3. Variables

QISU records for ED presentations with a principal diagnosis of an injury (ICD-10-AM S00-T98) were included in this study. Patient data collected through the QISU includes: cause of injury, mechanism of injury, place, and part of place (where the injury occurred), activity of the injured person, the main object or substance involved in the injury and human intent. The QISU is designed to capture level 2 National Data Set for Injury Surveillance (NDS-IS) data, which includes Major Injury Factor product codes in addition to routinely collected emergency data. QISU sites use a combination of text based and coded fields to enter data, depending of the patient management system used. Data were obtained from 1999 to 2014.

NHMD hospital separation records with a principal diagnosis of an injury (ICD-10-AM S00-T98) and an external cause code indicating the injury was the result of an accident (V00-X59) were included in this study. Patient data collected through the NHMD includes: patient demographics, diagnoses, external cause of injury, length of hospital stay, and inpatient hospital procedures undertaken. Data were obtained from July 2002 to June 2012.

The NCIS contains coded information as well as coronial files including autopsy, toxicology, police, and coroner reports. The NCIS captures data about injury fatalities Australia-wide using the International Classification of External Causes of Injury (ICECI) to describe injury causes. The ICECI, similar to NDS-IS, collects objects/substances in separate data fields to injury mechanisms. Cases were selected if they were coded with a Primary or Secondary Object code for any of the mandated products. Data were restricted to closed cases recorded on the NCIS system from 2004 to 2014.

### 2.4. Statistical Analysis

The data specifications and classification systems for the state-based injury surveillance emergency presentation data, national hospitalization data, and national coronial data systems were reviewed to identify relevant codes and data fields to identify opportunities for identification of any of the products with a mandatory safety standard. For only the product where data were available across two of the three systems, descriptive analysis was conducted to examine the frequency of presentations over time, age and sex patterns, and mechanism of injuries and types of injuries sustained. As the first aim was to investigate how effectively information regarding product-related injuries is able to be captured within Australian health and coronial data, the authors chose to only report the extent of injuries/deaths (the second aim) for those cases where at least two data sources captured data pertaining to these products. Statistical analysis was conducted using IBM Statistics for Windows, Version 22 (Armonk, NY, USA) [10].

### 2.5. Ethics Approval

This research study was approved by the University Human Research Ethics Committee (Approval number 1300000849) and by the Department of Justice Human Research Ethics Committee (for ethical approval to access the NCIS data, Approval number M0289). Access to data was approved by the Queensland Injury Surveillance Unit, the Australian Institute of Health and Welfare and the National Coronial Information System.

## 3. Results

### 3.1. Health and Coronial System Ability to Capture Mandatory Standard Products

#### 3.1.1. Queensland Injury Surveillance Unit (QISU)

Five of 26 products/product types with mandatory standards pertaining to child hazards are captured in NDS-IS Major Injury Factor codes (See Table 1). Product types are collected in separate data fields to injury mechanisms, enabling the capture of different ways in which children may injure themselves with products beyond what is specified in systems such as the ICD-10-AM. In addition to the major injury factor codes, QISU collects a free text injury description recorded by the triage nurse which can be used to search for products which are not captured by the NDS-IS coding system. While there are some caveats with using free text fields such as these (e.g., inconsistencies in recording, misspellings etc.), it does provide another avenue for identifying products involved in injury events beyond what is captured by coding systems.

#### 3.1.2. National Hospital Morbidity Database (NHMD)

Six of 26 products with mandatory standards related to children can be clearly identified for hospitalized injury cases using the ICD-10-AM external cause coding system (See Table 1). Further information is provided through ICD-10-AM activity codes which categorize the type activity the person was undertaking at the time of injury. One code (U70.0) pertains to athletic activities involving fitness equipment such as exercise bicycles and treadmills, however, given the lack of specificity of products within this classification, this code cannot be used to monitor injuries associated with specific exercise products.

#### 3.1.3. National Coronial Information System (NCIS)

Eleven of 26 products/product types with mandatory standards pertaining to child hazards are captured in the ICECI Object/Substance codes (See Table 1). Further detail is available in the reports included on the NCIS system including forensic pathologist, autopsy, police reports, and coroners findings.

#### 3.1.4. Summary

Table 1 provides a summary of all the relevant codes for each of the classification systems and data capture points for the 26 mandatory standard products for children. For 14 of the 26 products, there is no specific code to capture the product in ED, hospital, or fatality data. Only six products could be identified in at least two of the three data systems (baby walkers, bunk beds, cots, children’s nightwear (not available in QISU data), prams/strollers, and bicycles). While bicycles are captured in the coding system for all three systems, further analysis of the frequency of bicycle injuries in children will not be included in this paper as a large proportion of bicycle-related injuries are responded to by transport authorities not consumer product regulators and are therefore outside of the scope of this research. The products for which further analysis was conducted are baby walkers, bunk beds, cots, children’s nightwear, and prams/strollers.

### 3.2. Injuries Associated with Products for Which Mandatory Safety Standards Exist

#### 3.2.1. Baby Walkers

Between 1999 and 2014, there were 202 baby walker-related ED injury presentations for children aged four years and younger recorded in QISU data (See Table 2). Low-level falls accounted for 51% of baby walker-related injury presentations and falls with a drop of 1 m or more accounted for 42% of presentations (See Table 3). Of all recorded baby walker-related ED injury presentations, 82% were not admitted to hospital and as a result, would not be captured by hospitalization data.

Between July 2006 and June 2012, 41 children aged 9 years and younger were admitted to hospital across Australia due to falls from baby walkers (See Table 4). Of these admissions, almost half presented in Queensland (*n* = 20), followed by New South Wales (*n* = 13), South Australia (*n* = 4), Victoria (*n* = 3), and Western Australia (*n* = 2). Over half the sample (64%) were infants under 12 months of age, with males representing almost two-thirds (64%) of all cases. Head injuries were the main principal diagnosis accounting for 85% of hospital admissions (See Table 5). For 39 of the 42 cases the length of stay was only 1 day, while the remaining 3 stayed for between 2–4 days.

There was one baby walker-related death due to a head injury in a male infant aged 11 months. The small cell size prevents further detail around this case, however the circumstances around the incident showed that the cause of the injury was not directly related to the design or the use of the product, however, it was related to the physical presence of the product which indirectly caused the accident.

#### 3.2.2. Cots

Between 1999 and 2014, there were 535 cases of cot-related ED injury presentations for children aged 14 years and younger recorded in QISU data (See Table 2). Falls accounted for 79% of cot-related ED presentations, contact with a static object accounted for 13% presentations and cutting or piercing accounted for a further 2% of presentations (See Table 3). Of all cot-related ED presentations, 84% were not admitted to hospital.

There were 489 children aged nine years and younger admitted to hospital across Australia due to falls from cots between July 2006 and June 2012 (See Table 4). Approximately one quarter (23%) of cot-related injuries were for infants under 12 months of age and three quarters (75%) of injured children were aged 1–4 years. Males represented 54% of all cases. Head injuries accounted for 55% of hospital admissions, followed by fractures of the upper extremities (including forearm, shoulder, and upper arm fractures), which accounted for 36% of hospital admissions (See Table 5). The average length of stay was 1.6 days (SD 2.3).

There were 11 cot-related deaths recorded in the NCIS database from 2004 to 2014. More than half these cases (*n* = 6) occurred from 2006 to 2007, with the majority of cases (*n* = 7) being for females. The youngest age was 3 months old and the oldest was 17 months old with most cases (*n* = 10) aged over 5 months old. All cot-related deaths were related to threat to breathing, with 8 cases caused by entrapment and 3 cases due to positional asphyxiation. Amongst the entrapment cases, the circumstances included being entrapped between the mattress and the side of the cot (due to ill fitted mattress and due to missing part, which contributed to the cot side being insecure); entrapment between the side dropdown and the cot base, and being entrapped through a missing slat on the base of the cot.

#### 3.2.3. Prams

Between 1999 and 2014, there were 943 cases of cot-related ED injury presentations for children aged 14 years and younger recorded in QISU data (See Table 2). Falls accounted for 90% of ED presentations, contact with a static object accounted for 5% and other specified external causes accounted for 3% (See Table 3). Of all pram-related injury presentations, 81% were not admitted to hospital.

There were 708 children aged 14 years and younger admitted to hospital across Australia due to falls from prams in the six year period July 2006 to June 2012 (See Table 4). Approximately half (51%) pram-related injuries were for infants under 12 months of age, and 54% of all pram injuries were for males. Head injuries accounted for 90% of hospital admissions, with the greatest proportion (37%) attributed to unspecified injuries of the head (See Table 5). The average length of stay was 1.2 days (SD 1.5).

There were 6 pram-related deaths recorded in the NCIS database from 2004 to 2014 with the last case occurring in 2007. Four were males with the youngest being two weeks and oldest being 11 months. Circumstances included infants not being supervised or left to sleep in the pram in combination with straps were not being used leading to entrapments, positional asphyxiation, and prams rolling down embankment into rivers with subsequent drowning.

#### 3.2.4. Nightwear

Nightwear related ignitions could not be identified in QISU data due to limited code availability, and there were no coronial cases identified in the time period that recorded nightwear as the product involved in the fatality.

There were 57 children aged below 19 years admitted to hospital across Australia due to ignition/melting of nightwear in the six year period July 2002 to June 2012 (See Table 4). Of these, the largest number occurred in New South Wales (*n* = 16), followed by Victoria (*n* = 13), and South Australia (*n* = 12). Almost half (46%) were aged 5–9 years of age and 58% were male. Burns of the trunk were the main principal diagnosis, accounting for 33% of hospital admissions followed by burns of the hip and lower limb (accounting for 28%) (See Table 5). The average length of stay was 3.77 days (SD 6.7).

#### 3.2.5. Bunk Beds

Between 1999 and 2014, there were 1465 bunk bed-related ED injury presentations for children aged below 19 years recorded in QISU data (See Table 2). Falls from heights of more than one meter accounted for 80% of presentations, low-level falls accounted for 12% of presentations and contact with static objects accounted for 6% (See Table 3). Of all ED presentations for bunk bed-related injuries, 79% were not admitted to hospital and therefore, would not be captured in hospitalization data.

There were 2036 children aged under 19 years admitted to hospital across Australia due to injuries associated with bunk beds in the six year period July 2006 to June 2012 (See Table 4). The greatest proportion of cases were among children aged 0–4 years (44%), with males representing 56% of all cases. New South Wales had the highest number of admissions (*n* = 578) (though was the only State where the number of admitted cases reduced over time from 117 in 2006/07 to 80 in 2011/12), followed by Queensland (*n* = 555), and Victoria (*n* = 446). Fractures of the upper extremities (including forearm, shoulder and upper arm fractures) accounted for almost half (48%) the hospital admissions, followed by head injuries, which accounted for 39% of hospital admissions (See Table 5).

There were two bunk bed-related deaths recorded in the NCIS database between 2004 and 2014. However, the small cell sizes prevent further detail regarding these cases.

## 4. Discussion

This study examined the ability to capture information about injuries related to products with mandatory standards pertaining to hazards for children, and the trends and patterns of child injuries and fatalities associated with these products. There are limited injury surveillance data available to identify and monitor child injuries and fatalities related to mandated products, severely limiting the evidence base to assess the efficacy of these regulations. Determining whether or not mandatory standards are effective and ensuring that dangerous products are not ‘falling through the cracks’ and causing serious injuries/deaths is not possible under a surveillance system that does not capture complete data regarding products of concern. All three data sources, emergency data collection, hospitalization collections, and coronial data have mechanisms for providing feedback regarding additional categories requiring capture. Furthermore, as hospitals become increasingly digitalized, and detailed clinical notes become more accessible electronically, there is significant potential for data mining to identify products of concern that may not necessarily be captured in routine coding systems. Both of these approaches require product safety regulators to be engaged with relevant health information authorities to lodge their interest in data capture and provide expertise in regards to data interpretation.

In this study, an analysis was conducted of five mandated products that have available injury data for at least two of the three data systems: baby walkers, cots, prams, nightwear, and bunk beds. These will be described in more detail below.

Baby walkers have had a mandatory standard enforced since late 2002, with the standard covering stability standards, braking mechanisms to prevent falls down stairs, and information standards for warning labels [11]. However, 55% of emergency department presentations related to baby walkers were due to falls down stairs, and the majority of injuries were head injuries suggesting issues with the stability and braking mechanisms of baby walkers. Khambalia et al. [12] found a four-fold risk of falls down stairs for infants in baby walkers compared to infants not using a baby walker in their systematic review. It is not possible to identify whether the products in use were old/second hand baby walkers which do not comply with the 2002 mandatory standards, though it is unlikely that injuries in more recent years could be attributed to old products on the whole. Previous research from New South Wales (NSW) has shown children using baby walkers aged eight months or less to be at 2.5 times greater risk of experiencing head injury than older children [13]. This is consistent with the outcomes of this study, where the largest number of baby walker hospitalizations occurred among children aged less than one year. Further research from Victoria and South Australia shows that 23% of baby walker-related hospital admissions occur due to falls down stairs, 67% occur due to children losing stability while using a baby walker, and 46% occur from children reaching hazards other than steps while using a baby walker [14]. This range of injury circumstances may be reflected in the diverse injury mechanisms associated with baby walkers captured through this study, which include cuts, burns, and fractures.

The mandatory standard for household cots came into effect late 2005, with the standard focused on the height of sides and absence of footholds to prevent falls, the size of gaps to prevent entrapment, protrusions, and the strength of cots [15]. However, a quarter of the hospitalized falls from cots were for infants under 12 months of age, who would have limited climbing ability, bringing into question the adequacy of the height of the sides/footholds for preventing falls. Furthermore, 30% of the ED-related presentations related to cots were for mechanisms other than falls, including overexertion and cutting/piercing mechanisms. These points towards potential issues related to gaps/protrusions on cots. Previous research from QLD reports similar patterns of cot-related hospitalizations, with the majority of injuries associated with falls (78%) and resulting in injuries to the head (64%) [16]. This study reported similar hospital admission rates to a study conducted in Victoria, where 14% of ED presentations associated with cot injuries required hospitalization [17].

The current mandatory standard for prams and strollers came into effect on 1 July 2008, with the standard covering performance testing, design, construction, safety warnings and informative labels for prams and strollers [18]. However, falls from prams were the most common cause of product injuries among children, with around half of emergency presentations and admissions for babies under one year of age. This suggests disregard of adult supervision and use of safety straps as described in the safety warnings and informative labels. Similarly to this study, previous research from QLD reports falls to be the most common mechanism of injury associated with prams, with nearly 10% of pram injuries caused by children being tipped out of prams while travelling on escalators or while travelling down stairs [7]. This highlights the importance of safety strap use, which may need to be communicated more effectively to parents. Pram failure has been previously attributed to 2% of pram injury cases [7], however, it is uncertain whether pram failure is associated with old/second hand prams that do not comply with 2008 safety standards.

The mandatory standard for children’s nightwear came into effect on 15 February 2007, with the standard covering the style of garment, type of fabric used, and the burning behavior of the fabric [19]. The majority (61%) of nightwear-associated burns among children were to the hip, lower limb, or trunk, with children aged 5–9 years experiencing the greatest proportion (54%) of nightwear burn injuries. Similar observations from hospitalization data in the UK were associated with children wearing oversized adult clothing as nightwear, which is exempt from the mandatory standard [20]. There is limited research from Australia investigating burn injuries among children caused by nightwear. One study from NSW reports the highest proportion of all clothing related burn hospitalizations to be among boys aged 5–14 years, attributed to fire play and other risk taking behaviors [21]. In this study, boys experienced a significantly greater proportion of burn injures than girls, which may be associated with similar behavioral factors.

The mandatory standard for bunk beds came into effect on 7 April 2005. This standard sets out essential safety requirements for bunk beds and other elevated beds used in domestic situations, nurseries and institutions, and functional durability, stability, and performance criteria to meet these safety requirements, in order to reduce the likelihood of deaths and injuries to children. The mandatory standard aims to reduce the likelihood of a sleeping child rolling out (through the use of guard rails) and prevent asphyxiation through entrapment or snagging on elements of the bunk bed [22]. The bulk of the injuries that are occurring are to children well below the recommended age for use of bunk beds (similar to the findings of Khambalia et al. (2006) in their systematic review [12]), with injuries more likely to occur while the children are playing on the bunk bed than sleeping on the bunk bed. Hence, while the design standards may be contributing to a reduction in injuries occurring, behavioral elements around the use of bunk beds for play still need to be addressed. Previous research from QLD reports falls from bunk beds as the most common mechanism of injury (76%), followed by being hit with a ceiling fan while using a bunk bed (14%) [16]. The large proportion of ceiling fan injuries is likely to be attributed to children using bunk beds for unsafe play, which again may result from lacking parent supervision.

There are some limitations to this study. The identification of cases relies on the accurate coding of products by coders in the ED/hospital/coronial office and injury surveillance is not the primary role of this workforce, hence the data presented may underestimate the true extent of the problem. A proportion of cases that are counted in the QISU emergency presentation data were also admitted to hospital in Queensland, and hence counted in the hospitalization data as well, however, the hospitalization data is reported for the entire country so this number is only a small fraction of the cases presented in the results. Finally, hospital data are episode-based and it is not possible to account for multiple episodes of care for a patient (such as transfers to other facilities), however, to reduce this issue, cases with an admission mode of transfer were removed from analysis.

## 5. Conclusions

Product-related injuries cause a considerable number of child hospital presentations, admissions, and deaths in Australia. Despite existing mandatory standards for 26 products known to be hazardous to children, there is a lack of consistency in capturing cases of injuries associated with these products between health and coronial data. This prevents a thorough understanding of existing mandatory standard efficacy.

There is significant scope for improvement in current product injury recording practices in the health sector. Accurate and timely injury data are crucial to ensuring a prompt response to dangerous products available on the market, preventing further injuries and deaths. It is necessary to implement standardized methods to record details of product injury cases, moving away from open-text fields in administrative records to ensuring systems exist to uniformly capture injury caused by products within ED, hospital, and fatality data.

## Figures and Tables

**Table 1:** Summary of Mandatory Standard Products and relevant ED, hospital, and fatality codes.

Mandatory Standard Products	Hospitalizations (ICD-10-AM)	ED Injury Surveillance (NDS-IS)	Fatalities–Coronial System (ICECI)
**Children’s products**			
aquatic toys	N/A	N/A	N/A
baby bath aids	N/A	N/A	N/A
baby dummies	N/A	N/A	C.3.6.01.75 Pacifier, dummy
baby walkers	W02.8 Fall from baby walker	102 Baby walker	C.3.6.01.05 Baby walker
basketball rings/backboards	N/A	N/A	N/A
bunk beds	W06.0 Fall from bunk bed	202 Bunk beds	C.3.5.01.01 Bunk bed
child restraints	N/A	N/A	C.3.6.01.20 Baby or child car seat
folding cots	W06.2 Fall from cot	104 Cots	C.3.6.01.30 Cot, crib, baby bed
household cots			
moveable soccer goals	N/A	N/A	N/A
nightwear for children	X05 Ignition/melting of nightwear	N/A	C.3.9.01.30 Nightclothes, pajamas, nightwear, underwear etc.
prams and strollers	W02.7 Fall from pram/stroller	101 Baby pram, pusher, etc.	C.3.6.01.01 Baby pram, buggy, pusher, stroller, carriage
projectile toys	N/A	N/A	C.3.6.02.18 Other toy weapon or projectile toy
self-balancing scooters	N/A	N/A	N/A
swimming and flotation aids	N/A	N/A	N/A
toys containing lead	N/A	N/A	N/A
toys containing magnets	N/A	N/A	N/A
toys for up to 36 months	N/A	N/A	N/A
**General products**			
balloon blowing kits	N/A	N/A	N/A
bean bags	N/A	N/A	N/A
bicycle helmets	N/A	N/A	C.3.11.05.10 Helmet
bicycles	V10-V19 Pedal cyclist transport	549 Bicycle	C.3.1.01.05 Pedal cycle
exercise bicycles	N/A	N/A	N/A
novelty cigarette lighters	N/A	N/A	C.3.9.08.05 Lighter, match
portable swimming pools	N/A	N/A	N/A
treadmills	N/A	N/A	N/A

**Table 2:** Presentation and admission of children with a product-related fall injury by age and sex through a sample of Queensland emergency departments, from 1999 to 2014.

Age	Male		Female		Total	
	Presented at ED *n* (%)	Admitted *n* (%)	Presented at ED *n* (%)	Admitted *n* (%)	Presented at ED *n* (%)	Admitted *n* (%)
Fall injuries caused by baby walkers						
<1 year	111 (90)	20 (95)	69 (86)	13 (87)	180 (89)	33 (92)
1–4 years	11 (10)	1 (5)	11 (14)	2 (13)	22 (11)	3 (8)
Total	122 (100)	21 (100)	80 (100)	15 (100)	202 (100)	36 (100)
Fall injuries from cots						
<1 year	97 (34)	22 (40)	76 (31)	13 (41)	173 (33)	35 (40)
1–4 years	187 (65)	31 (56)	165 (67)	19 (59)	352 (66)	50 (57)
5–9 years	4 (1)	1 (2)	5 (2)	0 (0)	9 (1)	1 (1)
10–14 years	1 (0.3)	1 (2)	0 (0)	0 (0)	1 (0*)	1 (1)
Total	289 (100)	55 (100)	246 (100)	32 (100)	535 (100)	87 (100)
Fall injuries from prams						
<1 year	231 (49)	69 (69)	215 (45)	39 (49)	446 (47)	108 (60)
1–4 years	226 (48)	31 (31)	247 (52)	39 (49)	473 (50)	70 (30)
5–9 years	8 (2)	0 (0)	11 (2)	1 (2)	19 (2)	1 (1)
10–14 years	2 (1)	0 (0)	3 (1)	0 (0)	5 (1)	0 (0)
Total	467 (100)	100 (100)	476 (100)	79 (100)	943 (100)	179 (100)
Fall injuries from bunk beds						
<1 year	10 (1)	2 (2)	5 (0.7)	1 (1)	15 (1)	3 (1)
1–4 years	362 (46)	81 (46)	289 (43)	61 (47)	651 (44)	142 (46)
5–9 years	327 (42)	78 (43)	311 (46)	59 (46)	638 (44)	137 (45)
10–14 years	82 (10)	16 (9)	68 (10)	8 (6)	150 (10)	24 (8)
15–19 years	4 (1)	0 (0)	7 (1)	0 (0)	11 (1)	0 (0)
Total	785 (100)	177 (100)	680 (100)	129 (100)	1465 (100)	306 (100)

**Table 3:** Mechanism of product related injury for children presenting to a sample of Queensland emergency departments, from 1999 to 2014.

Injury Mechanism	Baby Walkers *n* (%)	Bunk Beds *n* (%)	Cots *n* (%)	Prams *n* (%)
Cutting; piercing object	2 (1)	10 (1)	11 (2)	9 (1)
Exposure to hot drink; food; water; other fluid; steam; gas; or vapor (includes scalds)	1 (0.4)	-	-	-
Fall-high (drop of 1 m or more)	85 (42)	1170 (80)	137 (26)	47 (5)
Fall-low (on same level; <1 m drop or no information on height)	104 (51)	174 (12)	284 (53)	800 (85)
Other or unspecified transport-related circumstance	-	-	-	3 (0.3)
Machinery	-	3 (0.2)	-	-
Pedestrian	-	-	-	2 (0.2)
Struck by or collision with object	5 (2)	87 (6)	72 (13)	51 (5)
Struck by or collision with person	3 (1)	8 (1)	1 (0.1)	5 (1)
Other specified external cause	2 (1)	13 (1)	30 (6)	24 (3)
Unspecified external cause	-	-	-	2 (0.2)
Total	202 (100)	1465 (100)	535 (100)	943 (100)

**Table 4:** Age and sex of children admitted to all Australian hospitals with a product-related injury, from July 2006 to June 2012 (and July 2002 to June 2012 for nightwear).

Age	Male		Female		Total
	*n*	%	*n*	%	*n*
Injury caused by baby walkers (July 2006 to June 2012)					
<1 year	17	63.0	10	66.7	27
1–4 years	10	37.0	3	20.0	13
5–9 years	0	0.0	2	13.3	2
Total	27	100	15	100	42
Injury caused by cot (July 2006 to June 2012)					
<1 year	61	23.1	52	23.1	113
1–4 years	201	76.1	166	73.8	367
5–9 years	2	0.8	7	3.1	9
Total	264	100	225	100	489
Injury caused by pram (July 2006 to June 2012)					
<1 year	208	54.3	152	46.8	360
1–4 years	170	44.4	164	50.5	334
5–9 years	4	1.0	9	2.8	13
10–14 years	1	0.3	0	0.0	1
Total	383	100	325	100	708
Injury caused by bunk bed (July 2006 to June 2012)					
0–4 years	489	42.7	405	45.5	894
5–9 years	497	43.4	394	44.3	891
10–14 years	141	12.3	76	8.5	217
15–19 years	19	1.7	15	1.7	34
Total	1146	100	890	100	2036
Injury caused by the ignition/melting of nightwear (July 2002 to June 2012)					
0–4 years	16	48.5	5	20.8	21
5–9 years	13	39.4	13	54.2	26
10–14 years	3	9.1	3	12.5	6
15–19 years	1	3.0	3	12.5	4
Total	33	100	24	100	57

**Table 5:** ICD-10 diagnosis codes of children admitted to all Australian hospitals with a product-related injury, from July 2006 to June 2012 (and July 2002 to June 2012 for nightwear).

Injury Diagnoses	Fall Injuries Caused by Baby Walkers *n* (%)	Fall Injuries from Cots *n* (%)	Fall Injuries from Prams *n* (%)	Fall Injuries from Bunk Beds *n* (%)	Burn Injuries Caused by Ignition/Melting of Nightwear *n* (%)
S00 Superficial injury of head	8 (20)	47 (10)	143 (20)	84 (4)	-
S01 Open wound of head	7 (17)	34 (7)	111 (16)	148 (7)	-
S02 Fracture of skull/facial bones	3 (7)	14 (3)	51 (7)	82 (4)	-
S03 Dislocation, sprain and strain of joints of head	-	-	16 (2)	-	-
S06 Intracranial injury	5 (12)	45 (9)	54 (8)	195 (10)	-
S09 Other/unspec injuries of head	13 (32)	126 (26)	263 (37)	288 (14)	-
S19 Other/unspec injuries of neck	-	-	-	22 (1)	-
S42 Fracture of shoulder/upper arm	1 (2)	68 (14)	17 (2)	312 (15)	-
S52 Fracture of forearm	2 (5)	106 (22)	22 (3)	675 (33)	-
S61 Open wound of wrist and hand	-	-	14 (2)	-	-
S72 Fracture of femur	2 (5)	19 (4)	7 (1)	54 (3)	-
S82 Fracture of lower leg, incl. ankle	-	6 (1)	-	52 (3)	--
T20 Burn of head and neck	-	-	-	-	7 (12)
T21 Burn of trunk	-	-	-	-	19 (33)
T22 Burn of shoulder and upper limb	-	-	-	-	10 (18)
T23 Burn of wrist and hand	-	-	-	-	4 (7)
T24 Burn of hip and lower limb	-	-	-	-	16 (28)
T25 Burn of ankle and foot	-	-	-	-	1 (2)
Other diagnoses	-	19 (4)	10 (1)	124 (6)	-
Total	41 (100)	489 (100)	708 (100)	2036 (100)	57 (100)
